# Water Level Decline in a Reservoir: Implications for Water Quality Variation and Pollution Source Identification

**DOI:** 10.3390/ijerph17072400

**Published:** 2020-04-01

**Authors:** Zixiong Wang, Tianxiang Wang, Xiaoli Liu, Suduan Hu, Lingxiao Ma, Xinguo Sun

**Affiliations:** 1 China Water Resources Pearl River Planning Surveying & Designing Co, Ltd., Guangzhou 510610, China; wzx@ahau.edu.cn; 2 School of Engineering, Anhui Agricultural University, Hefei 230036, China; xl123123@sina.com; 3 Dalian University of Technology, Institution of Water and Environment Research, Dalian 116024, China; husuduan@mail.dlut.edu.cn (S.H.); lingxiaoma@mail.dlut.edu.cn (L.M.); 4 Huaiyin Institute of Technology, Jiangsu Smart Factory Engineering Research Center, Huaian 223003, China; sunxinguo48144562@163.com

**Keywords:** low water level, Biliuhe reservoir, water quality, change, identification of pollution sources

## Abstract

Continuous water-level decline makes the changes of water quality in reservoirs more complicated. This paper uses trend analyses, wavelet analysis and principal component analysis-multiple linear regression to explore the changes and pollution sources affecting water quality during a period of continuous reservoir water level decline (from 65.37 m to 54.15 m), taking the Biliuhe reservoir as an example. The results showed that the change of water level of Biliuhe reservoir has a significant 13-year periodicity. The unusual water quality changes during the low water level period were as follows: total nitrogen continued to decrease. And iron was lower than its historical level. pH, total phosphorus, and ammonia nitrogen were higher than historical levels and fluctuated seasonally. Permanganate index increased as water level decreased after initial fluctuations. Dissolved oxygen was characterized by high content in winter and relatively low content in summer. The pollutant sources of non-point source pollution (PC1), sediment and groundwater pollution (PC2), atmospheric and production & domestic sewage (PC3), other sources of pollution (PC4) were identified. The main source of DO, pH, TP, TN, NH_4_-N, Fe and COD_Mn_ were respectively PC3 (42.13%), PC1 (47.67%), PC3 (47.62%), PC1 (29.75%), PC2 (47.01%), PC1 (56.97%) and PC2 (50%). It is concluded that the continuous decline of water level has a significant impact on the changes and pollution sources affecting water quality. Detailed experiments focusing on sediment pollution release flux, and biological action will be explored next.

## 1. Introduction

Water quality in reservoirs is influenced by external runoff and internal sediment pollutants [1]. For flood control and benefit promotion, reservoirs always drain a certain amount of water to maintain lower water levels before the flood season for downstream safety, and store water afterwards to guarantee future water supply and power generation. This operation is obviously seasonal [2]. Meanwhile, hydrological factors such as precipitation, runoff and storage capacity of reservoirs are changing dynamically under the influence of natural changes and artificial regulations. This leads to the variation of the hydraulic power, pollution input and environmental conditions in reservoirs, and further changes in water quality [3]. More attention should be paid to the impact of hydrometeorology on water quality and the change of pollution sources [4]. In a study of the Mahabad Dam reservoir in Iran, it was found that climate change could change the distribution and variation of TP in the reservoir [5]. Research in the Xin’anjiang Reservoir showed that water quality indexes were influenced by hydrological factors [6]. Investigation of NO_3_-N levels in six reservoirs of the middle Missouri River (USA) during drought, recovery, and flood periods indicated the NO_3_-N value was generally lower during the drought period [7]. Moreover, a study in Lake Rotorua showed climate variation had a major impact on nutrients and water quality [8]. Research in the Heihe Reservoir also verified that water quality indexes including DO, TP, Fe and Mn were influenced by seasonal variations [9]. Similarly, a numerical model simulation indicated that seasonal variations and reduced water flow would increase river eutrophication in the Loire River (France) [10]. Studies also showed that hydrology would result in changes in aquatic ecosystems [11], and increased inflows from major tributary rivers could impact the abundance and persistence of cyanobacterial blooms [12].

When a flood occurs, large amounts of pollutants in the basin are flushed into reservoirs with the runoff. As a result, the water becomes turbid. This intuitive impact on water quality has gained much attention [13]. Research indicated that a flood can increase the pollution load of reservoirs in a short time and can also produce considerable sediment pollution along with the successive deposition of pollutants into the reservoir [14]. Moreover, runoff and pollution load peaks occur earlier during higher intensity rainfall events [15]. The sediments entering a reservoir during one flood event can account for more than 0.5% of the storage capacity [16]. Correspondingly, the change in reservoir capacity have received more attention when the water level of the reservoir continued to decline, while the impact on water quality was often ignored. It is reported that electrical conductivity, organics (carbon, nitrogen and phosphorus), and chlorophyll a in the Tapacura reservoir were on the rise during dry season [17]; the turbidity of Poyang Lake rose in the period of low water level [18]. Actually, although the input of pollutants was reduced during low water level or dry periods, the internal sediment pollution was worse because lower water levels could contribute to the release of sediment pollutants [19]. A study in tropical reservoirs in the Brazilian semiarid region showed that water level reduction during an extended drought period contributed to water quality degradation due to high algal biomass and high turbidity [20]. Besides, the reservoirs, especially the water source reservoirs, must ensure the water supply during dry seasons, which accelerates the decline of water level [21]. Clearly, the dynamic changes of water quality and identification of pollution source in a reservoir with continuous decline of water level are interesting issues.

The Biliuhe reservoir is a multi-annual regulating reservoir. The water level would continue to decline in the period of dry season with low precipitation. Investigations showed that the water level of the Biliuhe reservoir declined from 65.37 m to 54.15 m from April 2014 to June 2015, a drop of water level equal to 1/3 of the water depth of a normal high-water level. This paper aims to explore the dynamic changes of water quality and identification of pollution sources in reservoirs undergoing a continuous decline of water level taking the Biliuhe reservoir as a case study.

## 2. Materials and Methods 

### 2.1. Dynamic Monitoring Program of Water Quality in Biliuhe Reservoir

**Figure 1:**
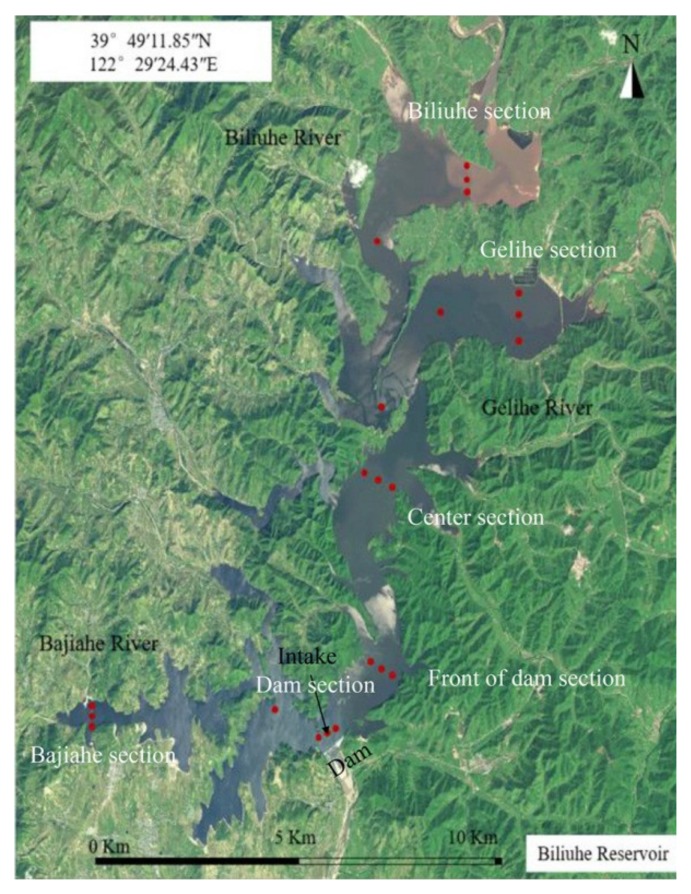
Distribution of monitoring sections and points in the Biliuhe reservoir.

The Biliuhe reservoir was built in 1983 with a drainage area of 2085 km^2^. The land use of the catchment is dominated by forest. Within the control basin, there are three cities, namely Zhuanghe, Pulandian and Wafangdian. The three major rivers are the Biliuhe River, Gelihe River and Bajiahe River. Sandaoling village, Guiyunhua town and Anbo town are at the entrance of the reservoir. The total annual average discharge of the reservoir is 16.7 m^3^/s. The length of the main dam is 708.5 m and the maximum height is 53.5 m. The total storage capacity is 9.34 × 10^8^ m^3^ and the water depth is about 30 m upstream of the dam. The Biliuhe reservoir is the main water source of Dalian. It has supplied water for Dalian since 1984. Now about 80% of the city’s total water supply comes from this reservoir. The Biliuhe reservoir located in the North temperate zone, an area with a humid climate. The four seasons are distinct, with an annual average temperature of 10.6 °C, while the annual average water temperature is 12.0 °C. The average annual precipitation of its control area is 742.8 mm and the corresponding annual runoff is 6.6 × 10^8^ m^3^. Based on the characteristics of the reservoir shape, water depth, etc., and in combination with the ‘Water and Wastewater Monitoring and Analysis Methods’ and the ‘Water Environmental Monitoring Specification’ (SL 219-98), a dynamic monitoring program for water quality in the Biliuhe reservoir was planned. Finally, six monitoring sections, four monitoring points and 22 monitoring vertical lines were set up in the main stream, two tributaries and reservoir areas (Figure 1). In addition, the monitoring points were dynamically adjusted during the field monitoring according to the water level changes. The adjustment rules are: (1) make it possible to collect all the samples of the planned monitoring points; (2) complete the monitoring program when the water level increases by adding unplanned monitoring points; (3) postpone the originally planned monitoring points when the water level declines.

The samples were taken once a month except in December, January and March, because the reservoir has a mixture of ice and water. The sampling at each monitoring point in each vertical line was collected according to the following principles. When the water depth was less than five meters, only one sample taken 0.5 m under the water level was required. When the depth was between five meters and ten meters, a water sample at a depth of 0.5 m above the reservoir bottom was also required. If the depth was more than 10 m, three water samples need to be collected at the depth of 0.5 m underwater, half of the depth and 0.5 m above the bottom, respectively. Significantly, the center of dam point was located around the intake of Biliuhe reservoir, so more attention should be paid on the sampling there. Sampling sites were each set five meters from the center of the dam to dynamically monitor the vertical change of water quality, and the number of water samples shall be increased or decreased according to the actual sampling conditions and water quality in practice.

The historical monitoring of Biliuhe reservoir showed total nitrogen was the major indicator that exceeded the standard for drinking water (GB3838–2002 (Table A1), Level Ⅲ). At the same time, the reservoir was at the risk of eutrophication. For further study, seven indicators were selected, including total nitrogen (TN), ammonia nitrogen (NH_4_-N), total phosphorus (TP), iron (Fe), permanganate index (COD_Mn_), dissolved oxygen (DO) and pH. A Niskin sampler was used to collect stratified water samples and the samples were all stored according to “Water Environment Monitoring Standard” (SL219-13). The selected indicators were all tested according to the national standard within the prescribed time. DO and pH were tested by the electrode method (multi-340i), TN was tested by the ammonium molybdate spectrophotometric method (HJ506–2009; HJ636–2012), TP was tested by the alkaline potassium persulfate digestion method (GB11893-89), NH_4_-N was tested by the Nessler reagent method (HJ535–2009), Fe was tested by flame atomic absorption spectrophotometry, and COD_Mn_ was tested by acid titration (GB11911-89; HJ/T100–2003). The quality assurance of all the tested samples was controlled in 95%.

### 2.2. Data Processing and Analysis

Based on field investigation and historical data, the characteristics of water quality under continuous low water level period were analyzed by means of equalization method, correlation analysis, wavelet analysis and PCA-MLR method in this paper.

#### 2.2.1. Equalization Method

Hydrology and water quality indicators are changing dynamically, so they are characterized by temporality and spatial diversity. To analyze the monthly changes of the water quality and hydrology in reservoir, the equation used is as follows [22]:

(1) $$\bar{x_{ij}}=\sum_{k=1}^{n}x_{jk}/n,$$

where $\bar{x_{ij}}$ is the average value of indicator *i* in the *j^th^* month, *x_jk_* is the *k^th^* observed value of indicator *i* in the *j^th^* month, *n* is the number of observed values of indicator *i* in the *j^th^* month. The mentioned historical average value below refers to the average value of the observed values from 1988 to 2012 of the corresponding months.

#### 2.2.2. Correlation Analysis Method

This paper analyzed the correlation of hydrology indicators and water quality indicators using the following equation [23]:

(2) $$r_{xy}=\frac{\sum_{i=1}^{n}\left(x_{i}-\bar{x}\right)\left(y_{i}-\bar{y}\right)}{\sqrt{\sum_{i=1}^{n}{\left(x_{i}-\bar{x}\right)}^{2}\sum_{i=1}^{n}{\left(y_{i}-\bar{y}\right)}^{2}}},$$

where *r* is the correlation coefficient, $\bar{x}$ and $\bar{y}$ are respectively the average value of water quality indicator *x* and *y*, *x_i_* and *y_i_* are the *i^th^* measured value and observed value of indicator *x* and *y*.

#### 2.2.3. Wavelet Analysis

The Morlet wavelet can be used to identify the periodicity of time series easily by calculating the wavelet variance, so wavelet analysis was used to calculate the periodicity of the water level in the Biliuhe reservoir. The main Morlet wavelet function was as follows [24]. The periodic analysis of a time series can be performed by drawing contour maps of wavelet coefficients:

(3) $$Var\left(a\right)=\int_{-\infty }^{+\infty }{\left|C_{x}\left(a,t\right)\right|}^{2}d\tau ,$$

where *C* is the wavelet coefficients. The wavelet variance can be used to determine the dominant period of the signal, so a higher variance represents a greater contribution to the signal.

#### 2.2.4. PCA-MLR Analysis Method

This paper adopted the PCA-MLR method to identify the pollution sources of each water quality indicator under low water level period conditions [25]. The PCA-MLR method uses the principal component analysis (PCA) method to reduce the dimensionality of each water quality indicator, thus less comprehensive indicators are formed. The extracted comprehensive indicators are not related to each other, and they can retain generally more than 75% or 80% of the original information. Then each principal component representing a source of pollution was identified by the factor load and theoretical analysis. Finally, the regression equation is obtained by conducting multiple linear regression analyses of the scores of the extracted principal components and the target water quality indicators. In the end, the contribution of each pollution source to the corresponding water quality indicator is obtained after normalization of the equation coefficients. This method is an effective way to preliminarily quantify the contribution of pollution sources owing to its simple calculation [26].

## 3. Results and Discussion

### 3.1. The Periodicity of Water Level in Biliuhe Reservoir

**Figure 2:**
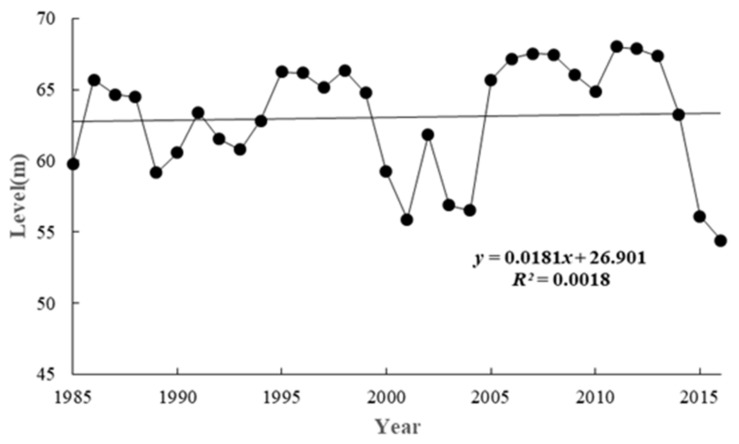
The annual variation of average water level in Biliuhe reservoir (1985–2016).

The annual variation of average water level in the Biliuhe reservoir from 1985 to 2016 is shown in Figure 2. The water level fluctuated periodically and showed an insignificant upward trend overall (Mann-Kendall-test Z = 1.086 < 1.96).

**Figure 3:**
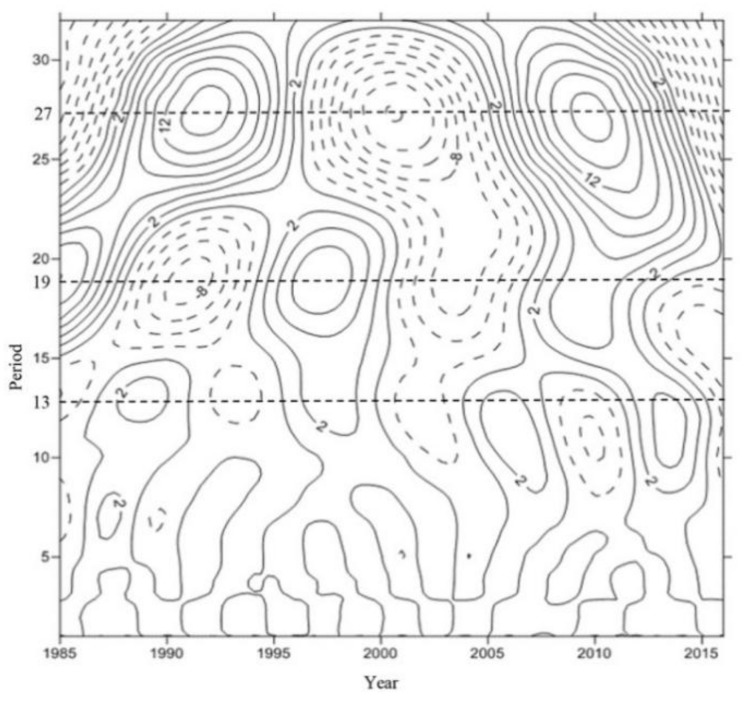
The wavelet analysis result.

Wavelet analysis is an effective method to process time-series data, especially those with non-stationary characteristics [27]. The water level records in Figure 2 show that from 1985 to 2015, it was highly non-stationary and non-linear. Then, the water level variation characteristics in the Biliuhe reservoir were further analyzed by a Morlet continuous wavelet function and the result is shown in Figure 3. The solid line indicates that the real part of the wavelet transform coefficient was positive, and the corresponding water level was rising [28], while the dotted line indicates that the real part of the wavelet transform coefficient was negative, and the corresponding water level declined.

The wavelet analysis showed that there were three water level periodicities, namely 26 to 29 year periodicities, 17 to 20 year periodicities and 10 to 14 year periodicities. The change process of wavelet variance with time scale of the Biliuhe reservoir sequences was further analyzed. There were three peaks which correspond to cycles of 13, 19 and 27 years. The periodicity scale of 19 years did not run through all the time and the contour of 27 year was not completely closed. The water level of Biliuhe reservoir had a periodicity variation of 13 years since it was built. In addition, in the study on the periodicity of precipitation in the Biliuhe reservoir, it is found that the precipitation had periodicity scales of 5–9 years and 15–18 years, and the corresponding peaks were 6 years and 17 years respectively. The interval of periodicity was close to that of the water level, but the peaks were different [29]. The changes of water level reflected the changes of precipitation, runoff and operation, which led to different water quality characteristics at different water levels. This paper pays more attention on the changes of water quality under the conditions of continuous water level decline. Further comparison of the periodicity with the actual water level changes showed that the water level of the Biliuhe reservoir from 1985–2001 (16 years) and 2001–2016 (12 years) showed a similar trend of rising-fluctuation (steady)-downward, and an obvious down trend from 1998–2001 and 2011–2016 (Figure 1).

### 3.2. Dynamic Changes of Hydrological Factors with Continuous Decline of Water Level

The changes of hydrological factors such as rainfall, runoff, water level, and storage capacity of reservoirs are important factors affecting water quality. As mentioned above, the water level in the Biliuhe reservoir had been significantly declining during 2011–2016. This paper selected the period of April 2014 to June 2015 to analyze the dynamic changes of hydrological factors with the continuous decline of water level.

**Figure 4:**
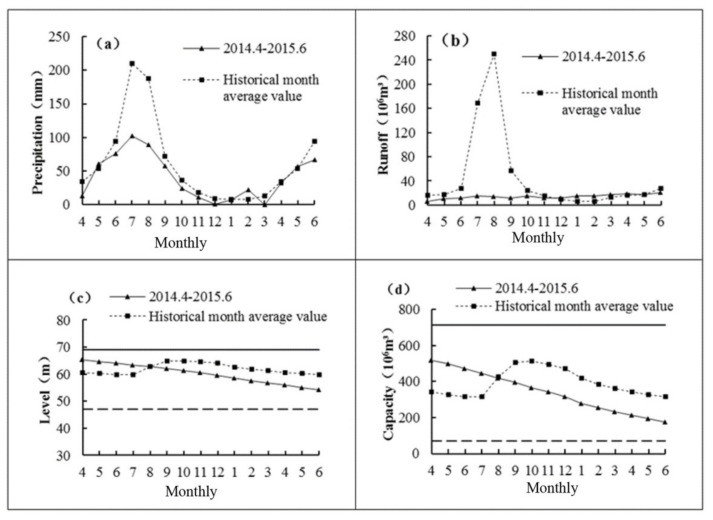
The monthly change of precipitation (**a**), runoff (**b**), water level (**c**), and capacity (**d**) of Biliuhe reservoir.

The Biliuhe reservoir is located at a semi-humid region, and the average annual precipitation in the region is 742.8 mm. The interannual variation is large and the precipitation is concentrated from July to August. The average total rainfall for the two months is 397.6 mm, accounting for 53.5% of the annual rainfall. Figure 4a shows that the precipitation from April 2014 to June 2015 was only 67% of the average precipitation for the same period. The precipitation in July and August 2014 was 102.2 mm and 88.6 mm, respectively, which accounted for 48.6% and 47.2% of the historical level. The direct impact of changes in precipitation is reflected in the runoff, water level, and storage capacity.

Similar to the change of precipitation, the average annual runoff of the Biliuhe reservoir also showed an obvious seasonal variation trend. As shown in Figure 4b, the historical average monthly runoff was the highest in the flood season in July and August. During the flood season, the average runoff was 4.18 × 10^8^ m^3^, which accounted for 68.8% of the annual runoff (6.08 × 10^8^ m^3^). Afterwards, the runoff gradually decreased as the dry season came. By the end of the winter, the runoff reached its minimum in December, January and February, which was mainly because the snow accumulated on the surface and could not generate runoff. When it came to March, as the ice and snow melted and the precipitation increased, the runoff began to increase until the arrival of the flood season. Then, the next cycle began. It can be seen from Figure 4b that the runoff into the reservoir during April 2014 to June 2015 was only 2.1 × 10^8^ m^3^, which was only 31% of the historical level. At the same time, in July and August in 2014, little runoff flowed into reservoir due to the small precipitation. The runoff in each month was only 9% and 6%, respectively, of the historical level, which was far below the historical level.

The Biliuhe reservoir is a multi-annual regulating reservoir. Discharge during the flood season, evaporation and water supply (electricity generation) were the main drainage ways. Natural runoff and diverted water from the Dahuofang reservoir constituted the inflow of Biliuhe reservoir. The specific operation rules are shown in Figure 4c. A common cycle is described as follows. The water level declined to the minimum before the flood season (June). Then, it continually increased during the flood season (from July to September) and reached its highest value in October. After that, it gradually declined until the following June because of the decreasing rainfall and continuous water supply.

In March 2014, the water level began to decline after the thaw, which was in line with the historical trend. However, the water level continued to decline from July and August 2014 because of low precipitation and insufficient water supply into the reservoir. In August 2014, the water level had fallen below the historical average level. What’s more, the water level continued to drop to 54.15 m in June 2015, which was 5.63 m lower than the average water level (59.78 m) in the same period, and was only 7.15 m higher than the dead water level (47 m).

Correspondingly, as shown in Figure 4d, the historical average storage capacity of the reservoir varied between 3.15 × 10^8^ m^3^ and 5.12 × 10^8^ m^3^, and the change trend was consistent with the water level trend. From July 2014, the storage capacity of the reservoir began to increase, and reached the maximum in October. Then, due to the reduction of rainfall and the duration of water use, the storage capacity began to decrease month by month. Similarly, the storage capacity of the reservoir had been declining during the period of low water level. In June 2015, it was only 1.76 × 10^8^ m^3^, which was close to the reservoir’s dead storage capacity of 0.7 × 10^8^ m^3^.

In the process of continuous decline of water level, precipitation was the driving factor of non-point source pollution. The reduction of rainfall restricted the input of runoff and non-point source pollutants, which was conducive to the improvement of reservoir water quality. Nevertheless, the self-purification capacity of the reservoir water body was decreased as well as the water environment capacity was reduced with the decline of water level and storage capacity [30]. In addition, the temperature, light and other conditions also changed with the continuous decline of water level. These changes directly affected the internal ecological environment of the reservoir, which promoted the release of pollutants from sediment and increased the load of the water [31]. Next, the changes of water quality with the continuous decline of water level were analyzed.

### 3.3. Dynamic Changes of Water Quality Indexes under Continuous Decline of Water Level

**Figure 5:**
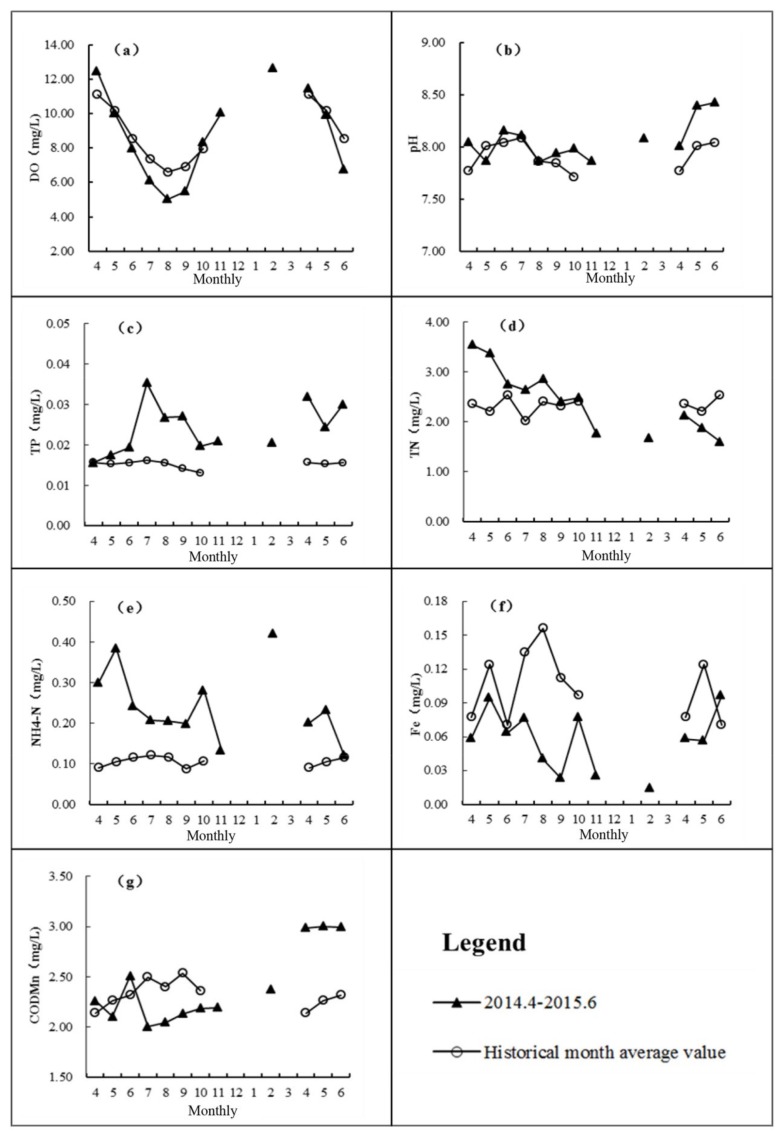
The monthly change of DO (**a**), pH (**b**), TP (**c**), TN (**d**), NH4-N (**e**), Fe (**f**), and CODMn (**g**) in Biliuhe reservoir.

Seven water quality indexes were selected to analyze the change of water quality, including DO, pH, TP, TN, NH_4_-N, Fe, and COD_Mn_. The data used for the mapping are average values calculated by Equation (1). The results are shown in Figure 5.

#### 3.3.1. Dissolved Oxygen (DO)

DO is an important indicator of water quality. The concentration of DO not only affects the life activities of aquatic organisms, but also plays a crucial role in the existence and the way of change of substances in the water body [32]. From the analysis of Figure 5a, it is found that the variation of DO in the Biliuhe reservoir during the low water level operation had little difference with that in the historical times. DO was always better than the water quality standard of Level III (≥5 mg/L, GB3838–2002), but in summer it was relatively poor. This is consistent with the findings of other researchers on the Gilgel Gibe reservoir and the Bakun reservoir [33,34].

The concentration of DO in a water body is related to the temperature, the content of oxygen-consuming substances, the re-oxygenation capacity, the composition of the sediments and aquatic organisms of the water body. The DO level is also in direct proportion to the temperature as high temperatures often lead to a decline of dissolved oxygen, in addition to the consumption of DO due to degradation of organic matter and reoxidation of reduced ions. The monitoring data showed that the DO in the reservoir had an opposite change trend to temperature, i.e., DO was lower in summer than in spring and autumn, and highest in winter. This was mainly related to the formation of a thermocline in summer that hindered the water-gas exchange within the water body [35,36]. In addition, the relatively high COD_Mn_ in summer also indicated that the DO consumption was higher than in other seasons. DO in summer had a corresponding decrease trend in water in low water level operation [37].

#### 3.3.2. pH

pH is also one of the basic water quality indicators. It can not only affect the direction of chemical reactions, but also can affect microbial activity. From Figure 5b, it was easy to find that the overall water quality of the reservoir was good. The pH was between 6 and 9 all the time, and the monthly difference was small in general.

Figure 5b shows a gradual increase from April to June and a gradual decrease from July to October. During the period from April 2014 to June 2015, the pH value was basically the same as that of historical periods, but higher than the historical level during July to August 2014 and April to June 2015. Similarly, the pH of the Muquém reservoir, Gilgel Gibe reservoir and Bakun reservoir were also higher during the dry period [33,38]. When the water level was low, the reservoir’s water environment capacity was small. The ability to resist external pollution was decreased, which made non-point sources have a greater impact on pH.

In addition, low water level conditions promote the release of phosphorus from the sediment, which results in an enhancement of algal activity, and finally affects the change of pH. The pH change in water is actually the result of acid-base equilibrium reactions. The reaction is mainly affected by pollution sources, algae and other aquatic plants. When there are no significant sources of pollution, algae and other aquatic plants will absorb CO_2_ in water and convert it into organics through photosynthesis. During this process, OH– will be released at the same time [39]. This series of reactions will damage the buffer system of CO_3_^2-^, HCO_3_^-^ and CO_2_ in the water, and then give rise to the increase of pH.

#### 3.3.3. Total Phosphorus (TP)

Phosphorus and nitrogen are both important elements for biological growth. Considering the nitrogen level is relatively high in China, phosphorus has gradually become a limiting factor of eutrophication [40]. Some studies suggested that water eutrophication may occur when TN and TP reached to 0.2 mg/L and 0.02 mg/L in water [40]. The average annual concentration of TN in the Biliuhe reservoir was 2.32 mg/L, and TP was 0.015 mg/L, indicating that phosphorus had become a limiting factor for the eutrophication in the Biliuhe reservoir. It can be seen from Figure 5c that the mean monitoring value of TP in the reservoir changed little overall, but it fluctuated greatly during low water level period (from April 2014 to June 2015). The change trend was consistent with the change of rainfall and runoff. The average TP concentration was 0.024 mg/L, which was significantly higher than the mean value over the same period (0.015 mg/L). It followed the same trend in the Bakun reservoir [34]. From April to July 2014, TP in the reservoir increased, and reached a maximum value of 0.035 mg/L in July. The analysis indicated that the pollutants carried by the runoff entered the reservoir and could not be fully degraded in July, which made the TP increase. From July to October, TP in the reservoir began to show a downward trend, because pollutants released from sediments increased due to the decrease of rainfall and water level. However, the discharge of production and domestic sewage in the basin caused the TP content of the reservoir still higher in the low water level period.

#### 3.3.4. Total Nitrogen (TN)

Nitrogen is an important nutrient in water bodies, and especially since the 1990s, the eutrophication caused by excessive nitrogen and phosphorus has been a major problem for lakes and reservoirs in China. It can be easily seen from Figure 5d that the TN in the Biliuhe reservoir exceeded the water quality standard (1 mg/L, GB3838–2002) all the time, and even exceeded the Level V (2 mg/L, GB3838–2002) most of the time. The mean value from April 2014 to June 2015 was 2.42 mg/L, which was a little higher than the annual average monitoring value of 2.33 mg/L.

As shown in Figure 5d, there was no significant change trend for the annual average monitored TN in the reservoir. However, in the flood season, the TN content of the reservoir was slightly higher. The increase may be related to the pollutants carried by rainfall in the flood season, or the release of nitrogen in the sediment to the water disturbed by rainfall runoff. In addition, atmospheric deposition of nitrogen with rainfall may also be an important influencing factor. Studies indicated that the wet deposition flux of TN in August of Dalian reached 4.42 kg/km^2^, which was proportional to rainfall [41].

The input and output of external pollution, adsorption and release of internal pollution, and self-purification of water body were the three factors affecting the content of TN in the reservoir. The TN showed a downward trend during the low water level period (from April 2014 to June 2015) of Biliuhe reservoir, which was related to the decreasing pollutants brought by the reducing rainfall and runoff and the self-purification of the water body. The same case was reported in the Bakun reservoir [34]. The slight increase of TN after a heavy rainfall in July also confirmed this viewpoint. The increase of TN in April 2015 may be caused by the effect of “turnover” of the reservoir and rainfall in early spring. After that, the TN content kept declining caused by the continuous decline of water level and precipitation, as well as the reduction of pollutants entered into the reservoir and self-purification of water body.

#### 3.3.5. Ammonia Nitrogen (NH_4_-N)

NH_4_-N, an important form of nitrogen, could be directly absorbed by algae and microorganisms in the water body. Therefore NH_4_-N was a main factor affecting the growth of algae and microorganisms. It can be seen form Figure 5e that the NH_4_-N in the Biliuhe reservoir was fine overall, and it was better than the Level II (≤0.5 mg/L, GB3838–2002) in most cases.

Regular annual monitoring data showed that the NH_4_-N in the reservoir changed little from month to month. The average value of each month was better than the Level I (≤0.15 mg/L, GB3838–2002). During the low water level period (from April 2014 to June 2015), the NH_4_-N fluctuated greatly with an average value of 0.24 mg/L. Due to the slow exchange of water in the reservoir, the NH_4_-N released from the sediment continuously accumulated in the reservoir, which lead to seriously excessive NH4-N content. Furthermore, the water environmental capacity decreased in the low water level period. The influence of domestic wastewater drainage on water quality was more prominent during this period. As a result, the content of NH_4_-N was obviously higher than the annual average monitoring value of 0.11 mg/L of the same period.

#### 3.3.6. Iron (Fe)

It can be easily seen from Figure 5f that the change trend of annual average Fe content was generally consistent with the rainfall runoff, i.e., the precipitation during the flood season was large and the content of Fe in the reservoir was relatively high. The content of Fe even exceeded the level 3 of water quality standard (GB3838–2002). During the low water level period (from April 2014 to June 2015), the content of Fe decreased, which might be related to the low water level, reduced rainfall, and the sedimentation of pollutants. Due to the small amount of precipitation in July and August 2014, there was almost no large inflow during this period, and the runoff into the reservoir was only 9% and 6% of the historical level respectively, which resulted in a reduction in the amount of non-point source pollutants during the flood season. Although the decrease of oxygen in summer will promote the release of Fe in the sediment [42], little amount of runoff and pollutants entered the reservoir, so the Fe in the flood season is reduced under the influence of the combined effects.

#### 3.3.7. Permanganate Index (COD_Mn_)

COD_Mn_ can reflect the comprehensive pollution of the water body. It is easy to see from Figure 5g that the COD_Mn_ of the Biliuhe reservoir was relatively low and changed little every month, of which the value was between Level I (2 mg/L, GB3838–2002) and Level II (4 mg/L, GB3838–2002). COD_Mn_ exceeded the drinking water standard all the time.

Judging from the trend of the annual average monitoring value of COD_Mn_, it slightly increased from April to July and changed little from July to October. It is considered that the pollution input increased with the rainfall runoff, at the same time, the water level and storage capacity of the reservoir also increased, which enlarged the water environment capacity. What’s more, it was also relatively insensitive to microbial activity, and the impact of temperature changes was reduced, so the COD_Mn_ remained a stable level from July to October.

With the gradual increase of rainfall, exogenous pollutants were input into the reservoir, and internal pollution was released from sediments caused by water disturbances [43]. Although the water level and the storage capacity gradually decreased, the overall water quality of Biliuhe reservoir was better than the level 3 of water quality standard (GB3838–2002). As a result, the COD_Mn_ showed a slight upward trend. The trend from April 2014 to June 2015 was similar to the historical average, but the average value was higher than the historical average. It is considered that the result is related to the decrease of rainfall, water level, and water environment capacity of the reservoir, as well as the pollution release of sediment.

### 3.4. Correlation Analysis of Hydrology and Water Quality Elements with Continuous Decline of Water Level

The results of correlation analysis by Statistical Program for Social Sciences (SPSS) are shown in Table 1. There was a positive correlation between DO concentration and precipitation, which was related to seasonal variation of precipitation. The temperature in summer with heavy rainfall was high, and high temperature led to low DO [44]. pH was negatively correlated with water level and storage capacity and was positively correlated with runoff and COD_Mn_. The decline of water level and storage capacity promoted the release of phosphorus in sediments and the propagation of algae, and further accelerated the consumption of carbon dioxide in water, which finally resulted in the rise of pH. At the same time, algae propagation made organics increase, causing the increase of COD_Mn_. TN was significantly negatively related with runoff, but positively related to water level and storage capacity, which was caused by less runoff and pollutants and denitrification of reservoir in low water level period [45]. TP was positively correlated with runoff, and it was because of the input runoff and hydraulic disturbance. NH_4_-N and Fe had no significant correlations with other indexes.

As shown above, V, P, R and L had good correlations with DO, pH, TP and COD under sustainable low water-level conditions.

### 3.5. Identification of Pollution Source in Reservoir with Continuous Decline of Water Level

In this paper, the monitoring water quality data (n = 414, April 2014 to June 2015) were used to identify the pollution sources of DO, pH, TP, TN, NH_4_-N, Fe and COD_Mn_. SPSS was used to conduct principal component analysis (PCA). The Kaiser-Meyer-Olkin (KMO) and Bartlett spherical test results were 0.560 and 543.07 (*p* = 0.00 < 0.05), respectively, which met the requirements of PCA for data and could be effectively carried out for principal component analysis. Table 2 shows the contribution of each principal component (PC). It can be seen that the cumulative contribution rate of the leading four PCs reached 80.78%, which could reflect the basic information of the original data, so the first four PCs were selected for further analysis.

The water quality index load shown in Table 3 represents the importance of each water quality indicator on the corresponding PC. The higher the absolute value of the water quality index load, the greater the influence on the corresponding principal component. Since the extracted principal components were mutually independent variables, the source of pollution represented by each principal component can be identified.

Based on the water quality index load and references [46], the analysis results are as follows: As shown in Table 2 and Table 3, PC1 accounted for 27.35% of the population variance. The index loads of DO, pH, TN, and COD_Mn_ were relatively larger, and these indices were all affected by rainfall runoff. Therefore, the first principal component was considered to represent non-point source pollution [47,48,49].

PC2 accounted for 24.41% of the population variance, in which NH_4_-N and Fe had a larger index loads. Sediment can release Fe, NH_4_-N and other pollutants. Especially under anaerobic conditions, the P and NH_4_-N that adsorbed on Fe(OH)_3_ were more easily resolved. However, the load of other index differs little, and the value was basically around 0.3–0.4, indicating that this pollution source had less and uniform influence on each index. This was consistent with the characteristics of groundwater pollution. Therefore, the second principal component was mainly represented by sediment pollution and groundwater pollution [50,51].

PC3 accounted for 19.37% of the population variance, in which DO, TN, and TP had higher index loadings. DO in water was mainly related to atmospheric recharge. TN and TP were affected by non-point source and sediment pollution as well as domestic sewage discharge. The analysis believed that it was mainly caused by atmospheric and production and domestic sewage in the basin [52,53].

PC4 accounted for 9.64% of the population variance. Except for TN, the load of each index was relatively close. Except for the mentioned pollution sources above, water quality was also influenced by the growing and migration of alga and benthos. Meanwhile, the decreased storage caused by water intake led to the increase of pollution release from sediment and atmospheric deposition, which indirectly influenced the water quality. This paper used the other pollution sources to represent the biological activities and water intake.

After identifying the pollution source of each principal component, the scores of the leading 4 principal components (PC1~PC4) and the water quality parameters were subjected to multiple linear regression (MLR). The results are seen in Table 4.

It can be seen from Table 4 that the determination coefficient of each indicator was fine, indicating that the reliability was higher. By normalizing the principal component coefficients of each regression equation, the contribution rate of the pollution sources to each indicator can be obtained.

As shown in Table 5, during the continuous low water level period, the DO in the Biliuhe reservoir was mainly affected by the atmospheric and production and domestic sewage (42.13%), non-point source pollution, sediment and groundwater pollution, and other sources of pollution respectively contribute 26.73%, 24.58% and 6.57%, which verified the viewpoint in Section 3.3.1. DO in water was mainly affected by atmosphere during low water level period, which was a main reason for its seasonal variation. The calculation result indicated that pH was mainly affected by non-point source pollution (47.67%) under low water level condition. Respect to the results in Section 3.3.2, the change of pH was influenced by many factors such as external pollution input, algal propagation and sediment release, which was in accordance with the PCA-MLR analysis. TP was mainly affected by production and domestic sewage (47.62%) and sediment and groundwater pollution (23.81%). It is agreed with the analysis of Section 3.3.3. As mentioned above, TP was influenced by low rainfall, which increased role of production and domestic sewage in TP pollution sources. The pollution sources of TN consist of non-point source pollution (29.75%), sediment and groundwater pollution (15.52%), atmospheric and production & domestic sewage (25.59%) and other sources of pollution (29.14%). As can be seen in Section 3.3.4, those sources affected the change of TN synthetically. NH_4_-N was mainly affected by sediment pollution (47.01%), which reflected the influence of sediment mentioned in Section 3.3.5. Fe mainly came from sediment and groundwater pollution (50%) and COD_Mn_ was mainly affected by non-point source pollution (56.97%). As mentioned in Section 3.3.6 and 3.3.7, low rainfall enhanced the influences of sediment and groundwater pollution and reduced the import of the non-point source, which verified the contribution rates were referable. The case study showed that the identification of pollution source based on PCA (APCS)-MLR can conveniently quantify the pollution source contribution rate of each indicator. Considering the difference of the selected indicators, the reliability of the results need to be further confirmed by more cases. However, it was still an effective method to determine the contribution rate of primary pollution source due to the advantage of simple calculation process.

## 4. Conclusions

The reservoir was affected by the natural-artificial regulation, and the water level was in a dynamic change. The continuous low water level process had a significant impact on water quality. This paper took the period of continuous low water level in the Biliuhe reservoir (April 2014 (65.37 m) to June 2015 (54.15 m)) as an example. The conclusions were as follows:

(1) The wavelet analysis showed that the water level periodicity of the Biliuhe reservoir was 13 years.

(2) In the low water level period, the on-site monitoring of the Biliuhe reservoir indicated that TN continued to decrease, Fe was lower than the historical level of the same period, pH, TP and NH_4_-N were higher than the historical levels and showed seasonal fluctuations, COD_Mn_ fluctuated first and then increased with the decline of water level, and DO showed the characteristics that are high in winter and low in summer with seasonal changes.

(3) DO during low water level was mainly influenced by atmospheric deposition and production & domestic sewage (42.13%). pH was largely affected by non-point source pollution (47.67%). Atmospheric and production & domestic sewage mainly (47.62%) explained the change of TP, while sediment pollution explained the change of NH_4_-N to a large extent (47.01%). For Fe and COD_Mn_, their changes were mainly attributed to the sediment and groundwater pollution (50%) and non-point source pollution (56.97%), respectively. It is concluded that the process of continuous decline of water level had a significant impact on the levels of water quality indicators. The PCA -MLR model, which has simple calculation process, can be used as an effective method to identify the pollution contribution rate to target indicator.

## Figures and Tables

**Table 1:** Correlation analysis of hydrology and water quality elements.

	V	P	R	L	DO	PH	TN	NH_4_-N	TP	Fe	COD_Mn_
V	1	0.189	−0.838^**^	0.996^**^	−0.159	−0.577^*^	0.889^**^	0.300	−0.416	0.287	−0.791^**^
P	0.189	1	0.272	0.153	−0.770^**^	0.209	0.175	−0.236	0.541	0.334	−0.109
R	−0.838^**^	0.272	1	−0.848^**^	−0.204	0.589^*^	−0.700^*^	−0.368	0.686^*^	0.071	0.678^*^
L	0.996^**^	0.153	−0.848^**^	1	−0.158	−0.628^*^	0.861^**^	0.301	−0.417	0.228	−0.830^**^
DO	−0.159	−0.770^**^	−0.204	−0.158	1	0.000	−0.033	0.526	−0.500	−0.081	0.277
PH	−0.577^*^	0.209	0.589^*^	−0.628^*^	0.000	1	−0.456	−0.226	0.249	0.172	0.748^**^
TN	0.889^**^	0.175	−0.700^*^	0.861^**^	−0.033	−0.456	1	0.371	−0.377	0.490	−0.547
NH_4_-N	0.300	−0.236	−0.368	0.301	0.526	−0.226	0.371	1	−0.552	0.094	−0.283
TP	−0.416	0.541	0.686^*^	−0.417	−0.500	0.249	−0.377	−0.552	1	0.000	0.234
Fe	0.287	0.334	.0071	0.228	−0.081	0.172	0.490	0.094	0.000	1	0.051
COD_Mn_	−0.791^**^	−0.109	0.678^*^	−0.830^**^	0.277	0.748^**^	−0.547	−0.283	0.234	0.051	1

V: storage capacity; P: precipitation; R: runoff; L: water level. Asterisk “*” indicates the correlation is significant (*p* < 0.05), and asterisk “**” indicates the correlation is highly significant (*p* < 0.01).

**Table 2:** Contribution of each principal component.

Principal Component	Contribution Rate (%)	Cumulative Contribution Rate (%)
1	27.35	27.35
2	24.41	51.76
3	19.37	71.14
4	9.64	80.78
5	7.12	87.90
6	6.57	94.47
7	5.53	100.00

**Table 3:** Water quality index load.

No.	Index	PC				Factor Commonality
		1	2	3	4	(Factor Load Square sum)
1	DO	0.421	0.387	0.664	−0.104	0.779
2	pH	0.746	0.312	0.152	0.354	0.802
3	TP	0.14	0.413	−0.749	0.332	0.862
4	TN	−0.555	0.29	0.477	0.544	0.915
5	NH4-N	−0.379	0.73	0.14	−0.305	0.789
6	Fe	−0.354	0.752	−0.275	−0.12	0.781
7	CODMn	0.765	0.327	−0.089	−0.161	0.725

**Table 4:** Multiple linear regression of each indicator.

**No.**	**Water Quality Parameters**	**Regression Equation**	**Parameter Test Associated Probability**	**Determination Coefficient**
1	DO	Z = 8.491 + 1.365F1 + 1.255F2 + 2.151F3 − 0.335F4	max{p} = 0.00 < 0.05	0.777
2	PH	Z = 8.034 + 0.337F1 + 0.141F2 + 0.069F3 + 0.160F4	max{p} = 0.00 < 0.05	0.802
3	TP	Z = 0.024 + 0.002F1 + 0.005F2 − 0.010F3 + 0.004F4	max{p} = 0.00 < 0.05	0.862
4	TN	Z = 2.502 − 0.343F1 + 0.179F2 + 0.295F3 + 0.336F4	max{p} = 0.00 < 0.05	0.915
5	NH_4_-N	Z = 0.239 − 0.057F1 + 0.110F2 + 0.021F3 − 0.046F4	max{p} = 0.00 < 0.05	0.789
6	COD_Mn_	Z = 2.323 + 0.392F1 + 0.168F2 − 0.046F3 − 0.082F4	max{p} = 0.00 < 0.05	0.725
7	Fe	Z = 0.051 − 0.029F1 + 0.061F2 − 0.022F3 − 0.010F4	max{p} = 0.00 < 0.05	0.781

**Table 5:** Contribution of pollution source to each pollutant.

**No.**	**Water Quality Index**	**PC1**	**PC2**	**PC3**	**PC4**	**Determination Coefficient**
		**Non-Point Source Pollution**	**Sediment and Groundwater Pollution**	**Atmospheric and Production and Domestic Sewage**	**Other Sources of Pollution**	
1	DO	26.73	24.58	42.13	6.56	0.779
2	pH	47.67	19.94	9.76	22.63	0.802
3	TP	9.52	23.81	47.62	19.05	0.862
4	TN	29.75	15.52	25.59	29.14	0.915
5	NH_4_-N	24.36	47.01	8.97	19.66	0.789
6	COD_Mn_	56.97	24.42	6.69	11.92	0.725
7	Fe	23.77	50.00	18.03	8.20	0.781

## Appendix A

**Table A1:** Water quality standard of surface water in China (GB3838–2002) mg/L.

Indicators	Level				
	1	2	3	4	5
PH	6–9			<6 or > 9	
DO≤	7.5	6	5	3	2
COD≤	15	15	20	30	40
NH4-N	0.15	0.5	1	1.5	2
TP≤	0.02	0.1	0.2	0.3	0.4
TN≤	0.2	0.05	1	1.5	2
Fe	≤0.3			>0.3	
